# Study on the Reactivity Activation of Coal Gangue for Efficient Utilization

**DOI:** 10.3390/ma16186321

**Published:** 2023-09-21

**Authors:** Yanshao Hu, Xiaoyan Han, Zuozheng Sun, Peng Jin, Keliang Li, Fuke Wang, Jinwei Gong

**Affiliations:** 1 State Key Laboratory of Coking Coal Resources Green Exploitation, China Pingmei Shenma Group, Pingdingshan 467000, China; huyanshao@126.com (Y.H.); jinpengxx@163.com (P.J.); 2 School of Civil Engineering and Transportation, North China University of Water Resources and Electric Power, Zhengzhou 450045, China; hanxiaoyan1@ncwu.edu.cn (X.H.);; 3 Institute of Materials Research and Engineering (IMRE), Agency for Science, Technology and Research, Singapore 138634, Singapore

**Keywords:** coal gangue, orthogonal test, genetic algorithm-back-propagation (GA-BP) model, strength activity index, activation mechanism

## Abstract

In this study, the research aim is to enhance the activity index of activated coal gangue and study its activation mechanism. The activation process of coal gangue was optimized through orthogonal tests, and the Back-Propagation (BP) neural network model was improved using a genetic algorithm. With the effects of grinding duration, calcination temperature, and calcination duration, the morphological changes and phase transformation processes of coal gangue were studied at the micro and meso levels to clarify the activation mechanism. The results indicated that the effect of calcination temperature on the strength activity index of coal gangue was most significant, followed by grinding duration and calcination duration. The potential activity of coal gangue can be effectively stimulated through mechanical and thermal activation, and the content of potential active minerals in coal gangue powders was also increased. The activation process of coal gangue for the optimal scheme was obtained as grinding at 76 min first and thermal treatment at 54 min at 749 °C. As the thermal activation under 950 °C, some unstable external hydroxyls, and internal hydroxyls in kaolinite from coal gangue were removed, the Al^Ⅵ^-O octahedron was destroyed, and kaolinite was transformed into spatially disordered metakaolinite with very high activity.

## 1. Introduction

Coal is one of the important non-renewable resources and related to global energy security. In recent years, coal consumption has accounted for over 50% of the one-time energy consumption in China [1]. Coal gangue is the main solid waste generated during the coal mining and washing process, which accounts for 15% to 20% of the coal production [2]. The cumulative emission of coal gangue has exceeded 7 billion tons in China [3], and it is still growing at a rapid rate of 0.5 billion tons per year [4]. A large amount of land has been occupied since coal gangue is piled in large quantities. The coal gangue spontaneously ignites when exposed to the air for a long time, causing fires and resulting in heavy economic losses and a large amount of toxic gases such as carbon monoxide (CO), hydrogen sulfide (H_2_S), and sulfur dioxide (SO_2_) to be released and pollute the environment [5,6,7]. In addition, the toxic heavy metals and other harmful substances seeped into the ground with rainwater, causing immeasurable ecological harm [8,9,10].

Faced with the present situation of the severe global resources environment situation, the demand for low-carbon and sustainable development, and the current shortage in construction and building materials, researchers have made many explorations into the efficient utilization of coal gangue as a substitute for the construction materials, such as backfilling in mining areas [11,12,13,14], as roadbed materials and concrete aggregates [15,16,17,18,19], preparing unburned bricks, etc. [20]. However, the utilization methods of coal gangue mentioned above have problems such as insufficient coal gangue consumption capacity, difficulty in controlling product quality, and low added value of products. There are many kinds of aluminosilicate minerals in coal gangue, but the chemical property of coal gangue is inert [21]. The key to using it as an alternative for preparing cementitious materials is to activate it to obtain the powder with a high pozzolanic reaction. Therefore, the methods of physical activation, thermal activation, chemical activation, and other means were used to activate the activity of coal gangue, and the preparation of low-carbon cementitious materials is currently an exploration of utilizing coal gangue as a building material resource [4,22,23].

The focus of current studies is to optimize the ratio of activated coal gangue powder and other types of industrial solid waste mixtures to improve their utilization rate [24]. With an activated coal gangue content of 75%, Yu et al. [20] have prepared bricks satisfying the requirements without high-temperature calcination by mixing slag and cement clinker. Huang et al. [25] and Koshy et al. [26] added blast furnace slag and quicklime to increase the calcium content of the coal gangue cementitious system, and the compressive strength of the mortar specimens after 28 days reached 57.83 MPa by chemical activation with sodium hydroxide and sodium silicate. Yang et al. [27] studied the compressive strength of low-carbon cementitious materials prepared from coal gangue and slag as raw materials activated by sodium hydroxide and sodium silicate, and the results indicated that the compressive strength still exceeded 39 MPa after 150 dry–wet cycles.

Thermal activation also positively disrupts the stable structure of coal gangue and stimulates its activity. Li et al. [28] and Zhang et al. [29] have studied the effects of the coal gangue powder sizes and activation temperatures on the performance of low carbon cementitious materials made from coal gangue and blast furnace slag activated by sodium hydroxide and sodium silicate. The results showed that the cementitious materials have the best performance when the fineness is 200 mesh and the activation temperature is 700 °C, but some amorphous active substances were transformed into stable crystalline states when the activation temperature exceeded 900 °C, which was not conducive to the polymerization process of the matrix. The mixtures of coal gangue and 8% CaO ground for 20 min were treated by hydrothermal treatment for 6 h first, calcination at 800 °C for 2 h and dry grind for 5 min then, and added it in the mixture of fly ash and blast furnace slag to prepare low carbon cementitious materials finally. It has been proved that the 28-day strength of the mortar specimens prepared by the above process were not be less than 42.5 MPa.

It should be noted that, subject to the material cost and test cycle, sometimes we need to use mathematical research methods to process these test data and optimize the process parameters by establishing predictive models. Chiya et al. [30] used the curing time of cement paste, water-binder ratio, and the content of microsilica and nanosilica as inputs to predict the mechanical properties of cement paste by constructing a multiple linear regression (MLR) model. Wang et al. [31] used an improved MLR based on Firefly Algorithm (FA) to predict the flexural strength of recycled concrete. After considering nine kinds of concrete preparation parameters, a good regression model was obtained.

However, there are many factors affecting the performance of building materials, and the relationship between various process parameters and material properties is not strictly linear, so it is difficult to predict the performance of building materials by using a simple MLR model [32]. BP neural network models have become another method to optimize the material production process in recent years because they can fully simulate the nonlinear relationship between each parameter and the response value in the material preparation process. Khademi et al. [33] used the BP neural network and MLR, respectively, to predict the compressive strength of recycled aggregate concrete with 14 parameters. The R^2^ of the BP model was 0.9226, while MLR was only 0.7456, indicating that the BP neural network had better prediction accuracy. Tanja et al. [34] adopted a BP neural network to model the relationship between cement content, water–cement ratio, and recycled aggregate replacement percentage of recycled brick aggregate concrete and its compressive strength to achieve reliable prediction of compressive strength. However, the BP neural network has problems with slow convergence and incorrect selection of optimal weights and thresholds, so many researchers will use an improved BP neural network to assist their research studies. Using 12 parameters as inputs, Wang et al. [35] predicted the unconfined compressive strength of marine soft clay stabilized by alkali residue and steel slag by constructing a BP neural network improved by Particle Swarm Optimization (PSO), and the R^2^ of the model could reach 0.9008. Ehsani et al. [36] predicted the fracture failure of concrete pavement using the BP neural network improved using the Simulated Annealing (SA) algorithm, and the R^2^ of the model could reach 0.976. Among many improved neural network algorithms, the BP neural network based on Genetic Algorithm (GA) doesn’t need an objective function to be continuous or differentiable and can search multiple objects at the same time, reducing the possibility of being trapped in local optimization [37]. Because it has better performance than a simple BP neural network [38], it is widely used in the field of property prediction of building materials. Compared with the BP neural network, using the GA-BP model to predict the compressive strength of concrete results in relatively small errors and stable changes in relative errors [39,40,41]. Therefore, this paper uses the GA-BP model to optimize the activation conditions of coal gangue and further improve the activation process of coal gangue through such a machine learning method.

This study uses grinding and calcination to activate coal gangue and uses orthogonal experiments combined with the GA-BP model to optimize the activation process of coal gangue and improve its activity index. Finally, the morphological changes and phase transitions of coal gangue under different activation conditions were characterized from a micro and meso perspective, and the activation mechanism of coal gangue was revealed through experiments such as laser particle size analyzer, X-ray fluorescence spectrometer, and scanning electron microscope. The results of this study aim to improve the activity of coal gangue as a supplementary cementitious material, therefore reducing cement consumption and carbon emissions. At the same time, it can effectively solve the accumulation of coal gangue waste and achieve waste resource utilization.

## 2. Experimental Program

### 2.1. Materials and the Activation Methods of Coal Gangue

#### 2.1.1. Materials

**Figure 1:**
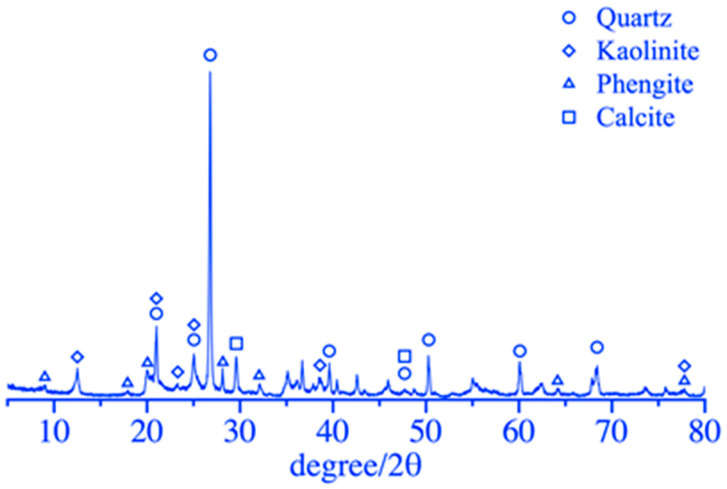
XRD pattern of raw coal gangue.

The 42.5 Ordinary Portland cement supplied by Zhucheng Yangchun Cement company limited (Zhucheng, China), the coal gangue in the mining area supplied by Pingmei Shenma Group (Pingdingshan, China), and ISO standard sand supplied by Xiamen Aisiou Standard Sand company limited (Xiamen, China) were used in this test. The chemical compositions of the cement and coal gangue are listed in Table 1. As listed in Table 1, the main chemical components of coal gangue were SiO_2_ and Al_2_O_3_, accounting for 86.09% of the total mass of coal gangue, while the content of CaO was only 4.58% and much smaller than that of cement in 59.12%. In addition, the loss on ignition of the coal gangue was 5.9 times that of the cement, indicating much more organic matter and residual carbon inside the coal gangue. The mineral composition of the coal gangue is shown in Figure 1, obtained using the X-ray diffraction (XRD) method. The main mineral phases in coal gangue were quartz and kaolinite. The diffraction peak of the quartz phase was sharp and clear, and the degree of crystallization was high. There were also some phengite and calcite phases. Therefore, appropriate mechanical and chemical activations were performed to effectively reduce the crystallinity and enhance the activity of coal gangue.

#### 2.1.2. The Activation Methods of Coal Gangue

The mechanical activation of coal gangue in this study was carried out using a ball mill, and the mass ratio of steel balls to coal gangue was 5:1. After ball milling, the coal gangue powder passing through the 0.212 mm square hole sieve was thermally activated using a muffle furnace. After the activation, the coal gangue was naturally cooled in the furnace. The entire activation process of coal gangue was performed by controlling the grinding duration, calcination temperature, and calcination duration. The effects of three factors (grinding duration, calcination temperature, and calcination duration) of the activation method on the activity of coal gangue were studied in this paper. 

### 2.2. Test Method

The morphological changes, phase transitions of activated coal gangue under different conditions, and the activation mechanism were studied through the microscopic and mesoscopic tests, as listed in Table 2.

The activity of the coal gangue was evaluated by measuring the compressive strength of the mortar. The design and compressive strength test of the cement mortar were based on China Standard GB/T 17671-2021 [42]. The significance of various factors on the activity of activated coal gangue and the preliminary optimized combination of activation conditions was studied using orthogonal tests. The cement mortars for the orthogonal tests were prepared by replacing 30% cement with activated coal gangue by weight. The three factors and three levels of the orthogonal tests are listed in Table 3. There are nine groups (CG-1~CG-9) of cement mortars listed in Table 4 for the orthogonal tests and one group of reference cement mortar (RE) without coal gangue in this study. Three specimens for each group with the size of 40 mm × 40 mm ×160 mm were produced with a 28-day curing period in a curing room at 20 ± 5 °C and over 95% relative humidity. The strength activity index (*H*_28_, %) was determined by Equation (1), according to China Standard GB/T 1596-2017 [43].

(1) $$H_{28}=\frac{R}{R_{0}}\times 100$$

where *R* and *R*_0_ are the 28-day compressive strength of the cement mortar using activated coal gangue and the reference group, respectively.

## 3. Results and Discussion

### 3.1. Orthogonal Test Results and Discussion

**Figure 2:**
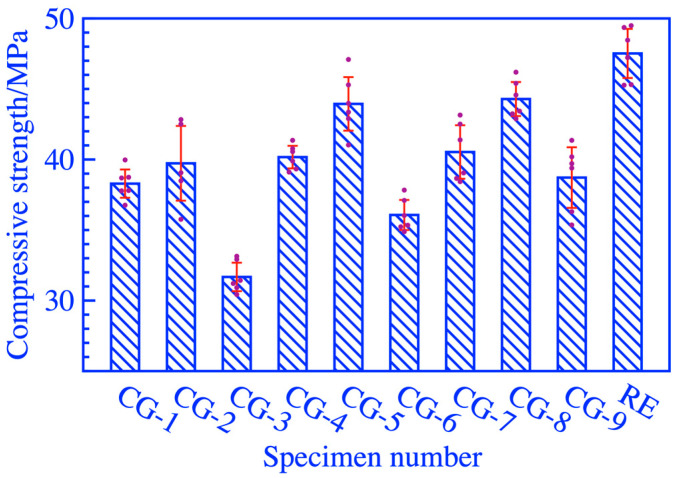
The compressive strength test results of cement mortar.

The 28-day compressive strength measured and activity index calculated with Equation (1) of the specimens in different factors and levels were shown in Figure 2 and Table 4. The dots represent multiple sets of parallel test data for the same formula, and the red lines represent the standard deviation of the data for it. The effects of grinding duration and calcination temperature on the compressive strength of cement mortar using activated coal gangue were significant, as shown in Figure 2. The optimal group of the orthogonal test groups was CG-8, and the grinding duration, calcination temperature, and calcination duration were 90 min, 750 °C, and 30 min, respectively. The 28-day compressive strength of RE is 44.29 MPa, and the activity index has reached 93.20%, which is 26.54% higher than CG-3, as listed in Table 4. Where k1, k2, and k3 are the average values at different levels under three different factors, and R is the average range of k values for each factor.

According to the test results, the calculated range of strength activity index to each factor in these three levels was listed in Table 4. For grinding duration, calcination temperature, and calcination duration, the range values of the strength activity index were 9.71%, 15.08%, and 1.74%, respectively. Therefore, the order of the influence level of the factors on the strength activity index from strong to weak was the calcination temperature, grinding duration, and calcination duration. The strength activity index increased with an increase in the activation temperature when the temperature was below 750 °C but decreased significantly when it exceeded 750 °C. The strength activity index increased with an increase in grinding duration (5~90 min), but the speed of the increase decreased significantly. The effect of the calcination duration (30~90 min) on the activity of coal gangue was the slightest. The range was only 1.74% and smaller than that of the error column (3.38%), indicating that within the selected calcination duration, changes in time cannot yield significant changes in the activity of activated coal gangue. The analysis of variance results for the strength activity index are listed in Table 5. The value of F corresponding to the calcination duration was only 0.27 and much smaller than F_0.20(2,2)_ = 9.00, indicating that the calcination duration has little effect on the activity of coal gangue, which was consistent with the range analysis results.

### 3.2. Establishment of GA-BP Model and Optimization of Activation

The significant impact of each activation process on the activity of coal gangue can be clarified through the orthogonal tests, but the related optimizations are based on combinations of established levels, which cannot be optimized in continuous intervals. In addition, only the optimization conditions can be determined through the orthogonal design, and the specific responses cannot be provided, which makes it difficult to evaluate the actual effects of the optimized activation process of coal gangue. Hereto, a neural network model was used to further optimize the activation process of coal gangue. Considering the possibility of interaction between calcination temperature and calcination duration, calcination duration was still used as an input variable in the modeling process. In order to reflect the impact of changes in process parameters on the strength of the specimens more intuitively, the 28-day compressive strength was selected as the response value. 

To avoid obtaining local optimal values through iterative calculation of neural networks due to limited sample size data in orthogonal tests, the following measures were adopted. First, two genetic algorithm modules were embedded in the main program of the neural network to form a GA-BP neural network, which iterated within randomly generated intervals to avoid falling into the local optima of a single interval. Then, two new sample points as the test set through tests were added to ensure the accuracy of the model. Though it was few in number of orthogonal data, directly using them as both the training and testing sets still led to data leakage during the training process of the neural network, greatly reducing the model’s anti-generalization ability. Therefore, the addition of these two new sample points above was very necessary. The activation process parameters of the two newly added sample points are listed in Table 6.

**Figure 3:**
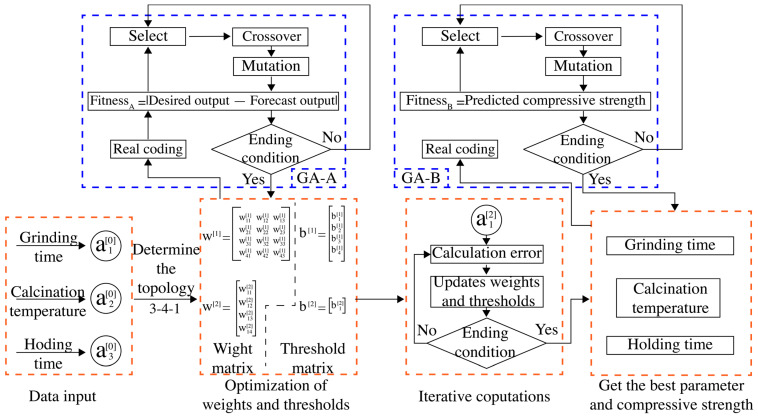
The establishment and operation of the GA-BP model.

The GA-BP model was established and operated as shown in Figure 3. The main program in the neural network was surrounded by the orange box, while the two different genetic algorithm modules (GA-A and GA-B) were surrounded by two blue boxes. The GA-A module was used to optimize the weights and thresholds of the neural network, while the GA-B module was used to globally search for the optimal activation process and corresponding 28-day compressive strength values after the establishment of the neural network prediction model. The model was established as follows.

(1) Normalization of data

The physical meanings and numerical differences of grinding duration, calcination temperature, and calcination duration are significant. To avoid the difficulties in model convergence and the significant errors of network prediction, Equation (2) was used to normalize these data into the values in 0~1.

(2) $$x^{\prime }{}_{k}=\frac{x_{k}-x_{\min}}{x_{\max}-x_{\min}}$$

where *x*_k_, *x*′_k_, *x*_min_, and *x*_max_ are data before normalization, data after normalization, and the minimum and maximum values in these data sequences for each activation process, respectively.

(2) The operating parameters and hidden layer nodes of the neural networks

In the neural network, activation parameters were used as the input parameters. A single hidden layer structure was used. Output nodes were for 28-day compressive strength prediction output. The maximum number of iterations was 1000. The learning rate was 0.1. The activation function was a sigmoid function. The reasonable range of values (2~12) for the number of hidden layer nodes was determined using Equations (3)–(5) [44], and the optimal number of hidden layer nodes was determined by a trial and error method to be four, with an error of 1.39 × 10^−7^.

(3) l<n−1

(4) $$l<\sqrt{m+n}+a$$

(5) $$l=log_{2}n$$

where *l*, *n*, and *m* are the node numbers of hidden layers, input layers, and output layers, and *a* is a constant between 1 and 10.

(3) The code, selection, crossover, and mutation methods of genetic algorithms

The total number of individuals within the operation matrix was 21 when GA-A was used to optimize the weight threshold. The GA-B encoding length was three, representing the values of three activation parameters. The roulette wheel method was used as the selection operation, shown in Equation (6). The fitness function determined by GA-A was the prediction error of the neural network; the smaller, the better. The roulette wheel rule defaulted to the individual with the highest calculation value as the optimal individual, so it was necessary to take the reciprocal of the fitness function of GA-A. The method used for crossover operation was real number crossover, and the mutation method was single point mutation [45].

(6) $$p_{i}=Q_{i}/\sum_{i=1}^{S}Q_{i}$$

where *p_i_* is the probability that the *i*-th individual will be selected, *Q_i_* is the fitness value, and *S* is the total number of the selected population.

(4) Determination of the genetic algorithm parameters

**Figure 4:**
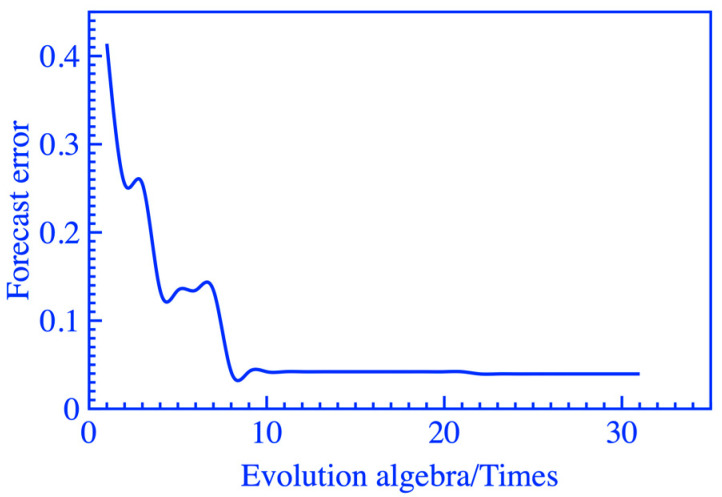
Iterative graph of GA-A.

**Figure 5:**
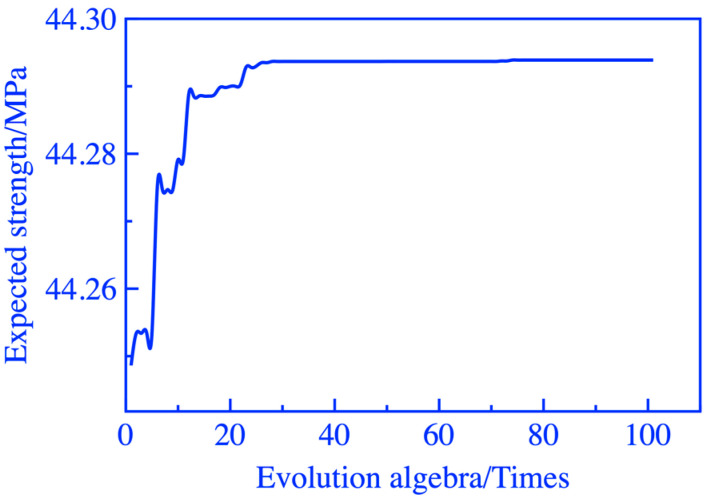
Iterative graph of GA-B.

The population, crossover probability, and mutation probability of the two genetic algorithm modules were 50, 0.3, and 0.1, respectively. Considering the speed of model operation and the reliability of the results, the number of iterations for GA-A and GA-B were set to 30 and 100, respectively. The convergence of fitness values is shown in Figure 4 and Figure 5.

The prediction results of the GA-BP model improved by the genetic algorithm and the traditional BP model were listed in Table 7, and the prediction accuracy of the GA-BP model was higher than that of the BP model. The activation process parameters of coal gangue for the optimal scheme obtained from the GA-BP model were 76 min, 749 °C, and 54 min for the grinding duration, calcination temperature, and calcination duration, respectively. The specific surface area of the coal gangue powder after grinding is 1287.55 m^2^/kg, and the predicted and measured 28-day compressive strengths were 44.29 MPa and 46.72 MPa, respectively, with an error of 5.20%, weight was w [1] = [1.5684, 2.2593, 0.4351, 1.0764, −2.8181, 0.4598, −2.6355, −0.2698, −2.0496, 1.7247, −2.3366, 0.5831], and w [2] = [−2.4970, 2.0319, −1.7904, −1.3782], biases were b [1] = [1.2971, 2.7980, −1.0025, −2.8049] and b [2] = [0.1836], respectively.

### 3.3. Activation Mechanism Analyses

#### 3.3.1. Analyses of Activation Mechanism of Ball Milling

**Figure 6:**
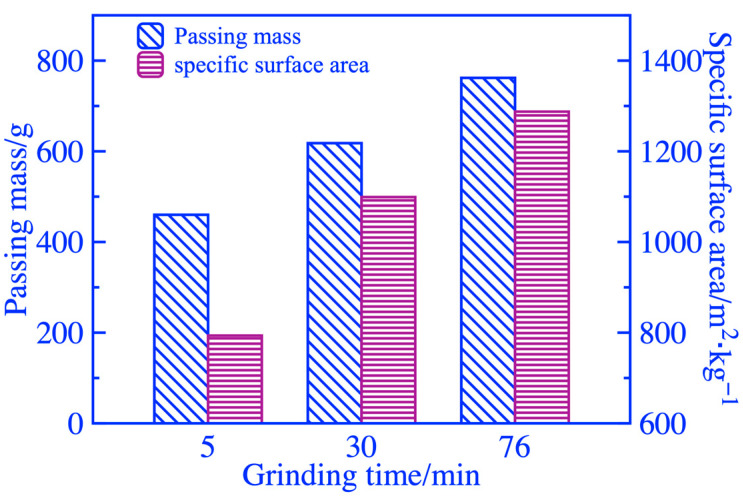
Wight screen residue and specific surface area at different grinding durations.

**Figure 7:**
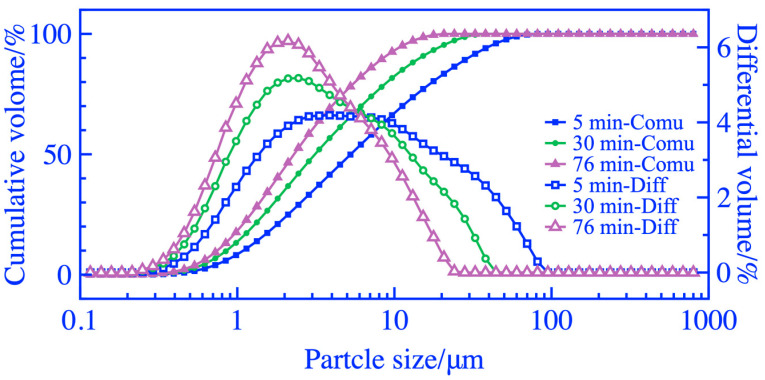
Particle size distribution of coal gangue.

**Figure 8:**
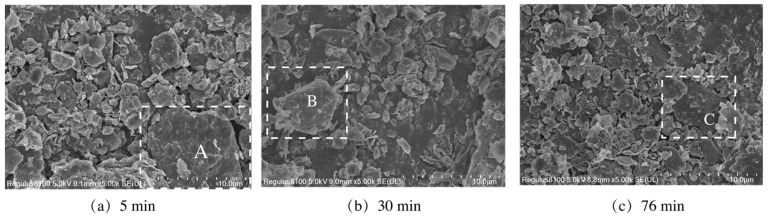
Microtopography of coal gangue after mechanical activation.

Keeping the initial feeding mass at 3 kg, the changes in the passing mass of 0.212 mm square hole sieves and the specific surface area of the coal gangue powders with grinding duration are shown in Figure 6. To increase the passing mass of square hole sieves and specific surface area, it is necessary to increase the grinding duration appropriately. Figure 7 shows the particle size distribution of coal gangue powder. When grinding duration of 5 min, there is a wide and relatively flat peak in the distribution curve of particle size range of 3~10 μm. The peak value of the curve continuously shifted forward, increased, and narrowed as the grinding duration increased, indicating an increase in fine-grained coal gangue and more concentrated particle size. As the microscopic morphology of coal gangue powders ground for 5, 30, and 76 min obtained by SEM in Figure 8, the maximum particle size of coal gangue decreased as the grinding duration increased. The sizes of the maximum particles A, B, and C in coal gangue powders ground for 5, 30, and 76 min were 12, 7, and 5 μm, respectively, and the powders within the size of 2~3 μm became increasingly concentrated. The above rules were consistent with the results of particle size analysis.

**Figure 9:**
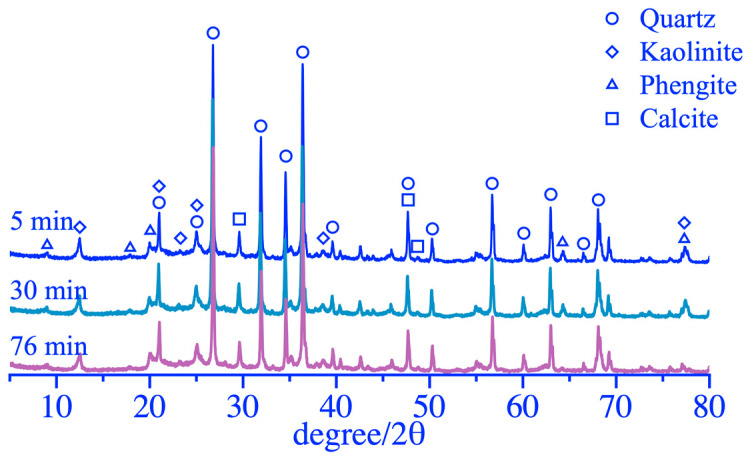
XRD patterns of coal gangue after mechanical activation.

**Figure 10:**
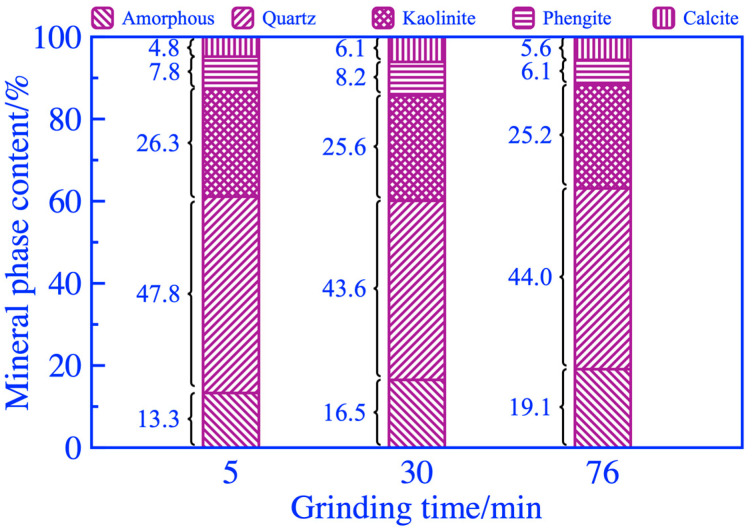
Mineral composition of coal gangue after mechanical activation.

**Figure 11:**
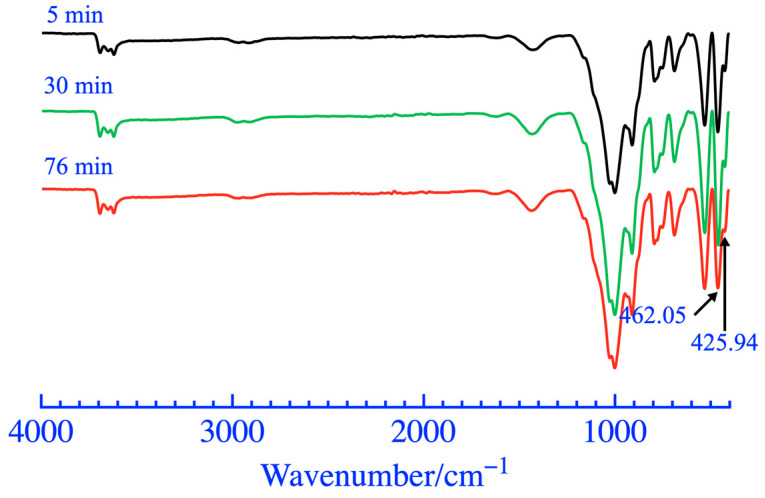
FT-IR patterns of coal gangue after mechanical activation.

The XRD analysis results and phase content of coal gangue powder are shown in Figure 9 and Figure 10, respectively. The diffraction peaks of most mineral phases did not show significant changes when the grinding duration was 5 and 30 min. When the grinding duration was 76 min, a significant reduction in the diffraction peak at a 2θ of 77°~78° was observed, caused by a decrease in the polysilicon phengite phase, as shown in Figure 10. In addition, as the grinding duration increased, there was a weak fading phenomenon in the diffraction peak of the kaolinite phase within 12°~13° of the 2θ, which was also caused by a reduction in the kaolinite phase and an increase in the amorphous phase. The FT-IR analysis results of coal gangue powders are shown in Figure 11. It exhibited a small amplitude fading for the Si-O-Si symmetric variable angle vibration peak at the point of 462.05 cm^−1^ when the grinding duration was 76 min. The small shoulder peak of Si-O-Al^VI^ bending vibration also slightly declined at 425.94 cm^−1^. This may be caused by the bond fracture of the Si-O-Si in part of the polysilicon phengite and the destruction of part of the kaolinite phase when it was ground for 76 min, as shown in Figure 9.

Overall, effective mechanical grinding was a feasible method to increase the content of potentially active minerals, amorphous active substances, and the number of active sites on the particle surface in the coal gangue powder and enhance the activity of coal gangue.

#### 3.3.2. Analyses of Thermal Activation Mechanism

**Figure 12:**
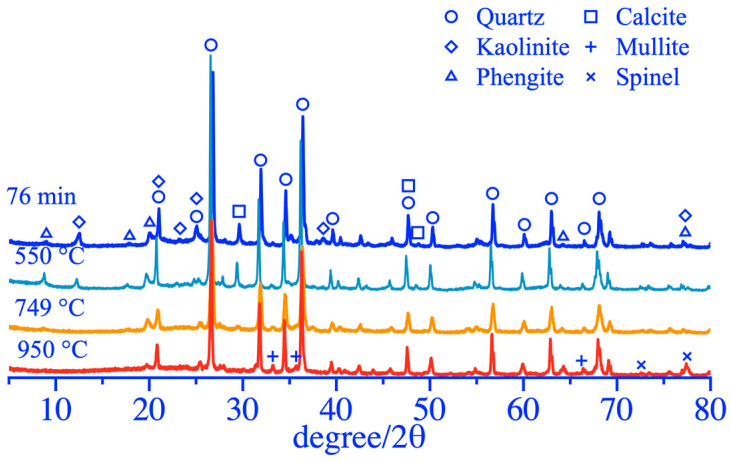
XRD patterns of coal gangue after thermal activation.

**Figure 13:**
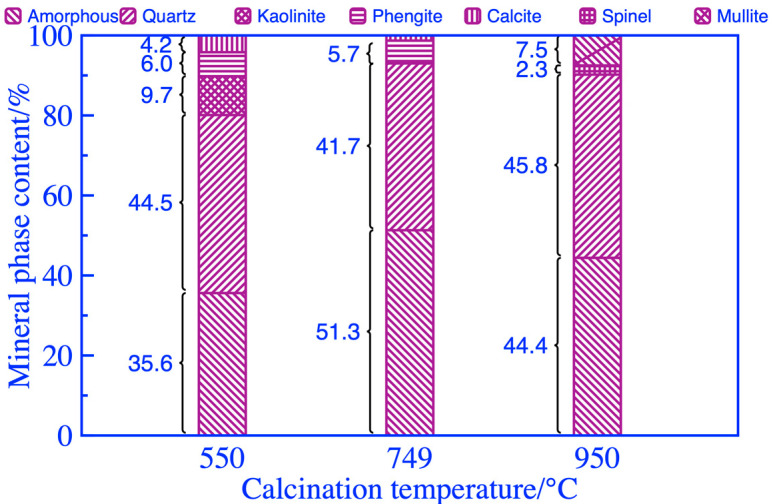
Mineral composition of coal gangue after thermal activation.

The thermal activation mechanism of coal gangue was studied that withstood the same grinding duration of 76 min and heat treatments at different calcination temperatures for 54 min; the obtained analysis results of XRD and phase content of coal gangue powder as shown in Figure 12 and Figure 13. The diffraction peaks of the kaolinite phase at 2θ of 12°~26° and 38.60° were significantly weakened after calcination at 550 °C and disappeared after calcination at 749 °C, and the destruction of the kaolinite phase during calcination resulted in an increasing content of the amorphous phase, as shown in Figure 13. The diffraction peaks of the calcite crystal phase at 2θ of 27°~29° and 49.60° exhibited no obvious change after calcination at 550 °C, and after calcination at 749 °C, the diffraction peaks almost disappeared with content of only 1.3% as shown in Figure 13. The diffraction peaks of the polysilicon phengite phase at 2θ of 9°~20° disappeared completely only after calcination at 950 °C. In addition, the quartz phase was extremely stable and did not suffer structural damage when calcined at 550~950 °C. After calcination at 950 °C, two new phases of mullite and spinel appeared in the coal gangue powder, and the diffraction peaks at 2θ of 66.20° and 77.50° showed some strengthening, resulting in a significant reduction in the amorphous phase and the activity of activated coal gangue powder as shown in Figure 13. Therefore, a better activation effect may not be obtained with the higher activation temperature, and the strength of the specimens prepared from coal gangue powder activated at 950 °C was relatively low.

**Figure 14:**
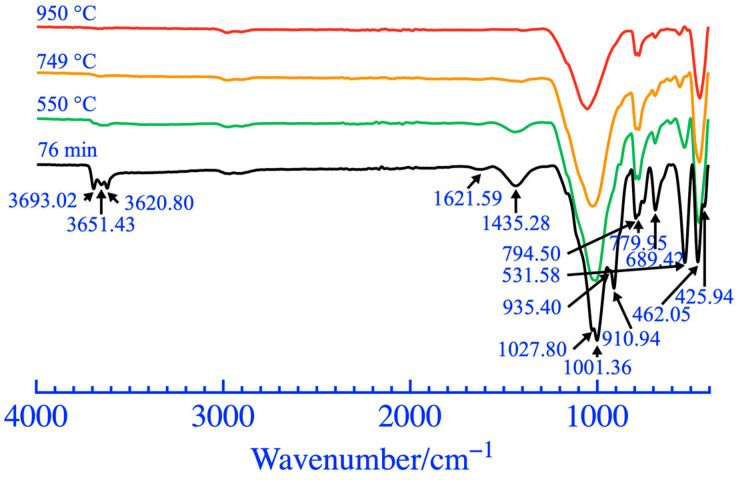
FT-IR patterns of coal gangue after thermal activation.

The FT-IR curve of coal gangue powders after thermal activations is shown in Figure 14. The phase structure of kaolinite was obviously destroyed after the activation at 749 °C, but a weak characteristic peak was still observed at 3600~3800 cm^−1^ at 749 °C. This was the weak characteristic peak of some hydroxyl groups in the polysilicon phengite that was difficult to separate, and it disappeared completely at 950 °C. In addition, the obtained changes in other phases and the analyses were consistent with the results in Figure 14, as listed in Table 8.

**Figure 15:**
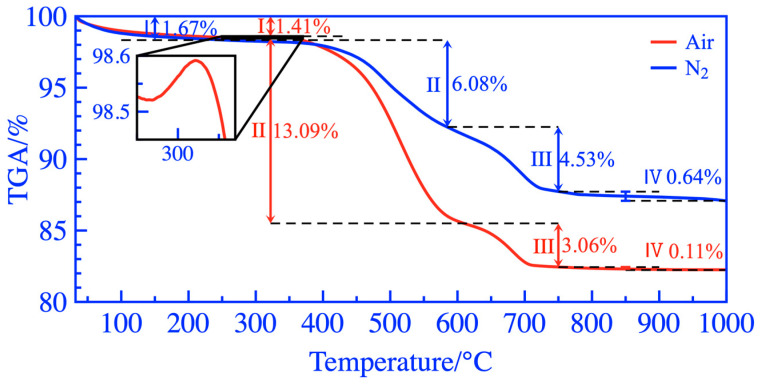
TGA curves in the air and N_2_.

**Figure 16:**
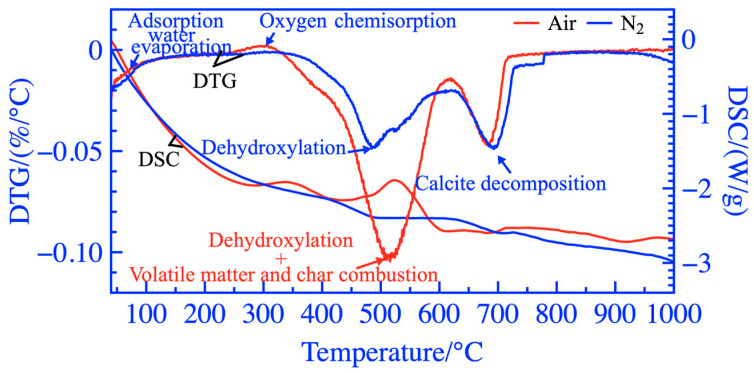
DTG-DSC curves in the air and N_2_.

The TGA and DSC test results of activated coal gangue powders under air and N_2_ are shown in Figure 15 and Figure 16, respectively. The weight of the coal gangue powder exhibited a loss of 17.67% during the heating process at 30~1000 °C in the air, while it only lost 12.92% weight in the N_2_ atmosphere. The coal gangue powder was in an endothermic state from 30 °C to 322 °C. At 262~322 °C, a weak peak was observed on the TGA curve with increased coal gangue powder mass, resulting in a clear exothermic peak near 330 °C on the DSC curve. During this process, the coal gangue powder adsorbs oxygen, accumulating sufficient oxygen for the subsequent combustion of residual carbon [48]. At 322~618 °C, the weight loss of coal gangue powder in the air was 7.01% higher than that in the N_2_ atmosphere, and the dehydroxylation endothermic peak of the kaolinite phase in DSC in the air was covered. According to the studies above, during the treatment of thermal activation, the hydroxyl removal of the kaolinite phase and the combustion of residual carbon and organic matter were important reasons for the significant improvement of the activity of coal gangue. At 618–750 °C, a small endothermic peak was observed on the DSC curve. The calcite decomposed to form CaO, which was converted into Ca(OH)_2_ during the hydration of cementitious material, thus further activating the activity of activated coal gangue. At 750–1000 °C, the weight loss during this stage was less than 1%. The stage is mainly the dehydroxylation reaction of the polysilicon phengite and the formation of a few mullite and spinel crystal phases; the endothermic and exothermic reactions occurred alternately so that no obvious endothermic and exothermic peaks were observed on the DSC curve.

**Figure 17:**
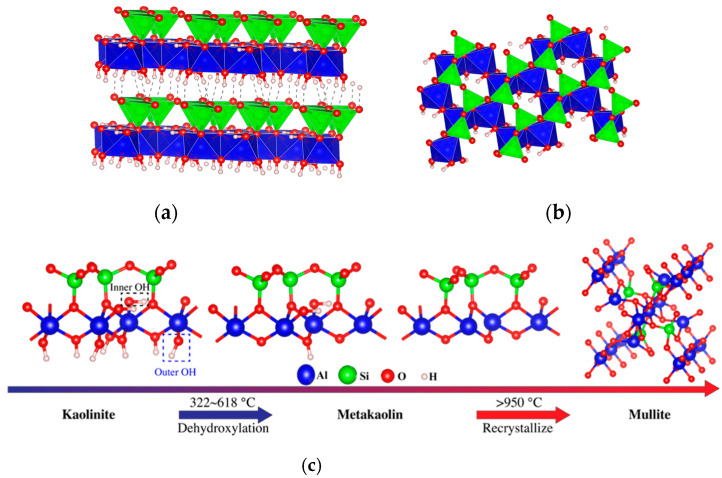
Crystal structure diagram and dehydroxylation diagram of kaolinite: (**a**) Crystal structure; (**b**) (001) Lattice plane; (**c**) Schematic diagram of dihydroxylation of kaolinite.

The thermal activation of coal gangue transformed the kaolinite phase into the amorphous metakaolinite phase. Figure 17 shows the crystal structure diagram and dehydroxylation diagram of kaolinite. The hydroxyl in kaolinite included the external hydroxyls at the edge of the double-layered structure and the internal hydroxyls within the double-layered structure interlayer. During the thermal activation, some unstable external hydroxyls were removed from the Al^Ⅵ^-O octahedron, and the coordination number of Al^3+^ became five. As the temperature continued to rise, the remaining externals and internal hydroxyls were removed, and the coordination number of Al^3+^ became four. The Al^Ⅵ^-O octahedron was destroyed, and the kaolinite was transformed into spatially disordered metakaolinite. The Al^3+^ with a coordination number of four in metakaolinite was unstable, so the coal gangue after the thermal activation at 322~618 °C exhibited very high activity. However, as the temperature continued to rise, the metakaolinite recrystallized into spinel and mullite, and its activity decreased significantly.

## 4. Conclusions

- The influence level order of the factors on the strength activity index from strong to weak was the calcination temperature, grinding duration, and calcination duration. The strength activity index increased with an increase in the activation temperature at 0~750 °C but decreased significantly when it exceeded 750 °C. The strength activity index increased in a significant decay rate with an increase in grinding duration.
- The prediction accuracy of the prediction results of the GA-BP model was higher than that of the BP model. The activation process parameters of coal gangue for the optimal scheme obtained from the GA-BP model were 76 min, 749 °C, and 54 min for the grinding duration, calcination temperature, and calcination duration, respectively, with a high strength activity index of 98.31%.
- The specific surface area of coal gangue powders was increased significantly by mechanical grinding. At the same time, the content of the amorphous phase and the number of active sites on the surface of coal gangue powder, as well as the content of active substances in coal gangue, were increased. Thus, the activity of the coal gangue was enhanced.
- The optimal temperature of the thermal activation was 750 °C. The thermal activation of coal gangue destroys the structure of the kaolinite phase and transforms it into an amorphous metakaolinite phase, in which the Al^3+^ with a coordination number of six was converted into active Al^3+^ with a coordination number of four. Thus, the activity of coal gangue has a significant improvement. At 618–750 °C, the calcite decomposed to form CaO and was converted into Ca(OH)_2_ during the hydration of cementitious material, which activated the coal gangue.

However, in the GA-BP model, the number of hidden layers and the learning rate value need to be determined using continuous attempts. In future research studies, more advanced algorithms can be used to further optimize some hyperparameter values to improve the performance of the model.

## Figures and Tables

**Table 1:** Chemical properties of cement and coal gangue (%).

	Content (%)	
Material	Cement	Coal Gangue
SiO_2_	26.53	60.44
Al_2_O_3_	4.03	25.65
CaO	59.12	4.58
Fe_2_O_3_	2.26	3.90
SO_3_	1.86	1.45
K_2_O	0.83	2.20
Others	5.37	1.78
Loss on ignition	3.08	14.25

**Table 2:** The microscopic and mesoscopic tests of the activated coal gangue powder.

No.	Test Instruments	Objectives
1	Panalytical Axios FAST X-ray Fluorescence Spectrometer (XRF)(PANalytical B.V., Almelo, The Netherland)	Determination of the chemical compositions of coal gangue
2	NKT2020-L Full Automatic Laser Particle Size Analyzer (Naikete, Jinan, China)	Analysis of particle size distribution of coal gangue powder
3	Regulus 8100 Field Emission Scanning Electron Microscopy (SEM) (Hitachi, Tokyo, Japan)	Observation of the microstructure of coal gangue powder and thermal-activated coal gangue powder
4	ALPHA Ⅱ Infrared Spectrometer (Bruker, MA, USA)	Fourier transform infrared spectroscopy (FT-IR) analysis of coal gangue powder after thermal activation
5	D8 ADVANCE X-ray Diffraction (XRD) (Bruker, MA, USA)	Determination of the phase composition and the amorphous phase content of the sample
6	STA 449C Simultaneous Thermal Analyzer(Netzsch, Selb, Germany)	Thermogravimetric analysis (TGA), Derivative thermogravimetric (DTG), and differential Differential scanning calorimetry (DSC) to clarify the combustion characteristics of coal gangue

**Table 3:** The factors and levels of the orthogonal tests.

Levels	Factor 1	Factor 2	Factor 3
	Grinding Duration (min)	Calcination Temperature (°C)	Calcination Duration (min)
1	5	550	30
2	30	750	60
3	90	950	120

**Table 4:** Orthogonal test results and range analysis.

No.	Grinding Duration (min)	Calcination Temperature (°C)	Calcination Duration (min)	Error	*H*_28_ (%)
CG-1	5	550	30	1	80.57
CG-2	5	750	60	2	83.61
CG-3	5	950	120	3	66.66
CG-4	30	550	60	3	84.53
CG-5	30	750	120	1	92.48
CG-6	30	950	30	2	75.90
CG-7	90	550	120	2	85.30
CG-8	90	750	30	3	93.20
CG-9	90	950	60	1	81.48
k1	76.95	83.47	83.22	84.84	
k2	84.30	89.76	83.21	81.60	
k3	86.66	74.68	81.48	81.46	
R	9.71	15.08	1.74	3.38	

**Table 5:** Variance analysis of strength activity index.

Factors	Freedom	Variance	*F*	*F*_α_	Significance
Grinding duration	2	77.01	7.02	F_0.01(2,2)_ = 99.01	△ ^1^
Calcination temperature	2	192.18	15.69	F_0.05(2,2)_ = 19.00	⨀ ^2^
Calcination duration	2	3.010	0.27	F_0.10(2,2)_ = 9.00	
Error	2	10.97		F_0.20(2,2)_ = 4.00	

Where △ ^1^ represents having an impact, and ⨀ ^2^ represents having a little impact.

**Table 6:** The activation process parameters of the two newly added sample points.

Points	Grinding Time (min)	Calcination Temperature (°C)	Calcination Duration (min)	Compressive Strength (MPa)
A	30	750	30	41.30
B	90	750	120	44.45

**Table 7:** Prediction error of different models.

Model	A/MPa	B/MPa	Error/%	Root Mean Square Error/MPa
BP	38.90	40.59	7.25	3.21
GA-BP	40.29	43.80	1.96	0.85

**Table 8:** The changes in the phases of the activated coal gangue powders are shown in Figure 14.

Wavenumber (cm^−1^)	Corresponding Substance	Calcination Temperature (°C)	Changes in Characteristic Peaks	Conclusion
1621.59	Adsorbed water [46]	550	Significant reduction	—
1435.28	Calcite [29]	550 749	Relatively stable Almost disappearance	—
1001.36 794.50 779.95 462.05 [47]	Quartz	550~950	No significant change	The quartz in coal gangue is extremely stable and difficult to decompose.
1027.80 689.42 531.58 425.94	Kaolinite	550 749	Significant reduction Almost disappearance	The calcined structure of kaolinite will be completely destroyed and transformed into an unstable amorphous phase in the range of 550~749 °C.

## Data Availability

Not applicable.
